# Pretreatment with IPA ameliorates colitis in mice: Colon transcriptome and fecal 16S amplicon profiling

**DOI:** 10.3389/fimmu.2022.1014881

**Published:** 2022-09-08

**Authors:** Yawei Fu, Hu Gao, Xiaohong Hou, Yue Chen, Kang Xu

**Affiliations:** ^1^Key Laboratory of Agro-Ecological Processes in Subtropical Region, Hunan Provincial Key Laboratory of Animal Nutritional Physiology and Metabolic Process, Institute of Subtropical Agriculture, Chinese Academy of Sciences, Changsha, China; ^2^Guangdong Laboratory for Lingnan Modern Agriculture, Guangzhou, China

**Keywords:** IPA, IBD, RNA-seq analysis, 16S rRNA sequencing, gut microbiota

## Abstract

3-Indolepropionic acid (IPA) is a tryptophan metabolite that has anti-inflammatory properties. The present study try to investigate the phylactic effects of IPA on dextran sodium sulfate (DSS)-induced colitis mice. The results showed that IPA pretreatment ameliorated the DSS-induced decrease in growth performance, and intestinal damage and enhanced immunity in mice. RNA-seq analysis of mouse colon samples revealed that the differentially expressed genes (DEGs) were mainly enriched in immune-related pathways. 16S rRNA sequencing showed that IPA pretreatment ameliorated DSS-induced colonic microbiota dysbiosis. Moreover, the expression levels of gut immune genes were positively correlated with the relative abundance of several probiotics, such as *Alloprevotella* and *Catenibacterium*. In conclusion, IPA alleviates DSS-induced acute colitis in mice by regulating inflammatory cytokines, balancing the colonic microbiota and modulating the expression of genes related to inflammation, which would also provide a theoretical basis for IPA as a strategy to improve intestinal health.

## Introduction

Inflammatory bowel disease (IBD) is common persistent incendiary infection, accompanied by a range of symptoms due to intestinal dysfunction, including diarrhea, rectal bleeding, abdominal pain and weight loss (1, 2). The incidence of IBD has become a major health issue in recent years (3, 4). Recent basic studies have identified risk factors for IBD that include malabsorption of selected nutrients (5) and the use of immunomodulators (6). Among the risk factors, dysfunctional immune regulation is considered to be one of the main causes of IBD (7). Therefore, normalizing the intestine is currently the main avenue of concern in the prevention and amelioration of the onset and progression of IBD.

Currently, most of the drugs targeting IBD are costly and have many side effects, and identifying new therapeutic approaches is crucial. Recent studies have revealed a crucial role of amino acid metabolites in the regulation of host immunometabolism (8, 9). In the intestine, the microbiota-derived tryptophan-metabolite IPA is a potential anti-inflammatory candidate molecule (10, 11). IPA played a role in maintaining the homeostasis of the intestinal environment in an indomethacin-induced intestinal injury model (12). Previous studies have shown that IPA can modulate intestinal microbiota composition and prevent gut dysbiosis and intestinal epithelial damage in rats fed a high-fat diet (13). On the other hand, IPA acts as an aromatic hydrocarbon receptor (AHR) ligand, and affects mouse intestinal barrier integrity by activating AHR signaling (14). However, it remains unclear whether regulation of the immune response is the key mechanism by which IPA alleviates colitis.

In this study, we investigated the effects of IPA on colonic inflammation and the composition of gut microbiota in a DSS-induced colitis mouse model. Our findings can provide a theoretical basis for IPA as a nutritional intervention to improve animal intestinal health and nutrition.

## Materials and methods

### Ethics statement

All experimental animals used in this study were treated humanely, following the Animal Welfare Committee of the Institute of Subtropical Agriculture, Chinese Academy of Sciences, Changsha, China.

### Experimental animals and tissue samples

Eight-week-old male C57BL/6 mice were randomly divided into 3 groups (9 animals for each group) as follows: the CON group, mice were fed normal drinking water; the DSS group, mice were fed 3% DSS in drinking water from Day 6 to Day 11) as the IBD model (15); and the DSS + IPA group, IPA(Sigma, CAS 830-96-6);at doses of 200 mg/kg body weight/day dissolving in 0.5% arboxymethyl cellulose sodium salt (CMC) was administered to mice by oral gavage and 3% DSS in the drinking water from Day 6 to Day 11. All mice were given free access to food and drinking water. On Day 12, the mice were euthanized, and samples were collected for subsequent analysis.

### Histological examination of the colon

Colon tissue samples were settled in 10% impartial buffered formalin and paraffin implanted, cut into tissue areas, and recolored with hematoxylin and eosin (16). Subsequently, the colons were assessed by histological examination (HE). Images of the samples were acquired by microscopy.

### Detection of immunoglobulins and cytokines in the colon and serum

Blood were collected from mouse eyes employing a serum separator tube and serum was separated through centrifugation(3,000 rpm, 15 minutes) (17). The contents of immunoglobulin A (IgA), interleukin 1beta (IL-1β), interleukin 4 (IL-4), interleukin 6 (IL-6), interleukin 10 (IL-10), interferon-γ (IFN-γ), and tumor necrosis factor alpha (TNF-α) in the serum and colon were determined using mouse -specific ELISA kits (Meimian Industry Co., Ltd, Jiangsu, China), according to the manufacturer’s instructions (18).

### 16S rRNA sequencing

The colonic contents of the CON, DSS, and ID groups were selected according to the preliminary results, and genomic DNA was extracted with the cetyl trimethyl ammonium bromide/sodium dodecyl sulfate (CTAB/SDS) method (19). Sequencing libraries were produced using the TruSeq^®^ DNA PCR-Free Test Planning Pack (Illumina, USA) following the manufacturer’s proposals and record codes were added (20). The Qubit@ 2.0 Fluorometer (Thermo Scientific) and the Agilent Bioanalyzer 2100 system were used to assess the library’s quality (21). Finally, the constructed libraries were quantified by Qubit and Q-PCR, and after the libraries were qualified, they were sequenced on the machine using NovaSeq 6000. diversity was applied to analyze the complexity of species diversity for a sample through 6 indices, including observed-species, Chao1, Shannon, Simpson, ACE, and good-coverage (22). Linear discriminant analysis (LDA) and effect size (LEfSe) analysis were used to detect bacterial taxa that differed significantly in abundance between groups (*P* value < 0.05; LDA score > 4) based on the nonparametric Kruskal-Wallis rank sum (23).

### Transcriptome sequencing

Colon samples from three individuals in each of the three groups (designated biological replicates) were subjected to high-throughput transcriptome sequencing as described previously. RNA sequencing data were processed using Trimmomatic, differentially expressed genes (DEGs) were identified using DESeq R package functions estimate size factors and nbinom test, and enrichment(GO and KEGG pathway);was performed based on the hypergeometric distribution (24). In addition, the DEGs expression pattern was visualized and analyzed by Short Time-series Expression Miner (STEM) software (25).

### Real-time PCR analysis

To verify the RNA-Seq data, six immune-related DEGs were randomly selected and assessed by real-time quantitative PCR (qRT–PCR). Primers were designed using the Primer5 and *β-actin* gene was used as the reference gene. RT-qPCR was performed with the miScript SYBR Green PCR kit (Thermo) and measured with the Roche LightCycler 480II. Each reaction was performed three times and calculated relative gene expression levels using a comparative CT method (referred to as the 2^−ΔΔCT^ method) (26).

### Association analysis of microbial and DEGs

As module 6 screened by the stem analysis was determined to be significant, we selected the DEGs enriched in module 6 and performed Spearman correlation analysis with the genus-level colon microbes (Top100). Co-expression networks were constructed in Cytoscape software using Spearman correlation coefficients (r ≥ 0.8, *P* < 0.05) and network analysis was performed (27).

### Statistical analysis

The results were expressed as mean ± SEM (standard errors of means) and analyzed by one-way ANOVA using SPSS (version 18.0, USA), and differences were considered significant at *P* value <0.05.

## Results

### Effects of IPA extract on DSS-induced colitis symptoms in mice

To investigate the potential phylactic effect of IPA on colitis inflammation, we studied its effect on colitis using the DSS- induced colitis model. As shown in **Table 1**, DSS- treated mice exhibited greater body weight loss and increased colonic weight with shortened colon lengths, while IPA pretreatment largely attenuated the effect of DSS, suggesting that IPA offered a more effective strategy for preventing colitis.

**Table 1:** Effects of IPA pretreatment on the growth performance of DSS-induced mice.

Group	Final body weight (g)	Colonic weight (g)	Colon lengths (cm)
CON	29.93 ± 0.93	0.63 ± 0.07^ab^	7.00 ± 0.32^a^
DSS	27.40 ± 0.95	0.74 ± 0.05^a^	6.36 ± 0.41^b^
ID	27.47 ± 0.57	0.53 ± 0.05^b^	6.77 ± 0.24^ab^

All values are expressed as the mean ± SEM (n = 9). a, b Means within a column with no common superscripts differ significantly (*P* < 0.05).

**Figure 1:**
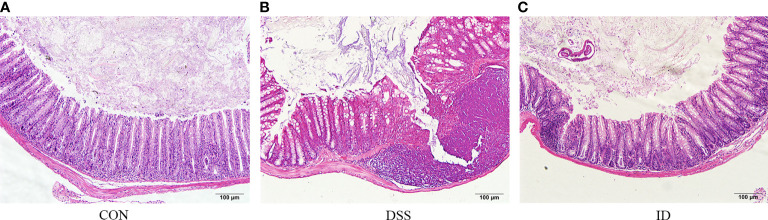
IPA alleviates DSS-induced colon epithelial damage. Hematoxylin and eosin-stained sections of colons from the CON group **(A)**, DSS group **(B)** and ID group **(C)** (scale bar = 100).

To observe the colonic pathological damage by DSS and ameliorated by IPA, we compared the pathological damage to the colon in the CON, DSS, and ID groups. Histological examination of colon tissue in the CON group revealed normal structure and regular morphology (**Figure 1A**). The DSS group developed apparent inflammation characterized by incomplete colonic structure (**Figure 1B**). Colon tissue from IPA treated mice showed mainly intact colon histology, with reduced signs of inflammation compared to the DSS group (**Figure 1C**). Taken together, these results demonstrate a protective effect for IPA in alleviating DSS-induced intestinal damage.

### IPA ameliorates the inflammatory response and increases immunity in DSS-induced mice

**Figure 2:**
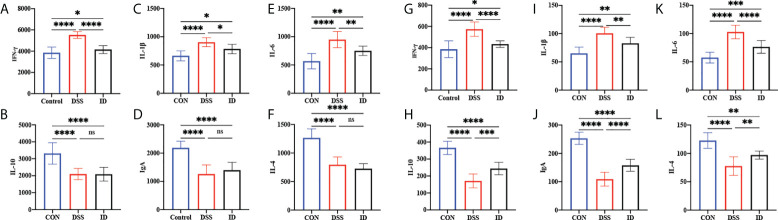
Effects of IPA pretreatment on inflammatory cytokines and immunoglobulins of the serum and colon in DSS-induced colitis mice. The colon was analyzed for the cytokines IFN-γ **(A)**, IL-10 **(B)**, IL-1β **(C)**, IgA **(D)**, IL-6 **(E)**, and IL-4 **(F)**. The serum was analyzed for cytokines IFN-γ **(G)**, IL-10 **(H)**, IL-1β **(I)**, IgA **(J)**, IL-6 **(K)**, and IL-4 **(L)**. All values are expressed as the mean ± SEM (n = 9). *P*>0.05 (ns), *P*<0.05 (*), *P*<0.03 (**), *P*<0.01 (***), *P*<0.0001 (****).

To assess the effect of IPA on the production of inflammatory cytokines and immunoglobulins linked to DSS-induced colitis, ELISA was performed on colonic tissues and serum from different treatment groups (**Figure 2**). The colon contents of the proinflammatory cytokines (IFN-γ, IL-1β, IL-6, and TNF-α); in colon of the DSS group were higher than the CON group, and the colon contents of the anti-inflammatory cytokines (IL-4 and IL-10) in the DSS group were lower than the CON group, while the opposite trend was observed in the CON and ID groups. The serum and colon contents of IgA were significantly lower in the DSS group than CON group, whereas the contents of IgA were significantly higher in the ID group than the DSS group.

### Effect of IPA on gene expression under DSS-induced inflammation in the colon

**Figure 3:**
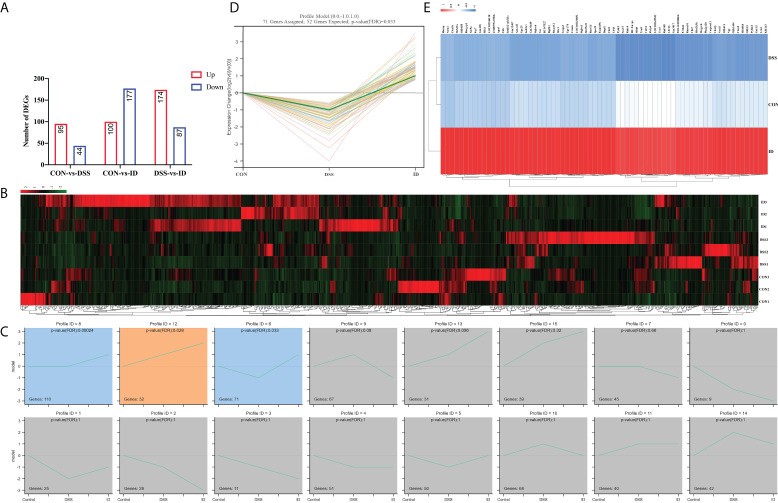
Regulation of colonic genes by IPA in DSS-induced colitis mice. **(A)** RNA-Seq analysis reveals DEGs among different groups. Red and blue represent the number of upregulated and downregulated genes, respectively. **(B)** Hierarchical clustering heatmap of different samples based on identified DEGs. **(C)** Modules in different colors represent different expression patterns that were significantly enriched by STEM analysis (nonsignificant modules in grey). **(D)** Trend graph of all genes under profile 6, where the thick green curves indicate the expression trend across all genes in a module. **(E)** The heatmap of genes from profile 6.

To investigate the possible mechanism by which IPA attenuated DSS-induced colitis, we performed transcriptome sequencing of colon tissues. After bioinformatics analysis, 139 (44 upregulated and 95 downregulated) DEGs, 277 (100 upregulated and 177 downregulated) DEGs and 261 (87 upregulated and 174 downregulated) DEGs were detected in the CON vs. DSS groups, CON vs. ID groups and the ID vs. DSS groups, respectively (**Figure 3A**; **Table S1**). To evaluate the DGE expression patterns, gene expression clustering was performed, and the results are presented in a heatmap (**Figure 3B**). Subsequently, we performed DEG STEM analysis to screen a total of 3 significant modules and plotted significant module trends and clustered heatmaps (**Figures 3C–E**; **Table S2**).

### Functional enrichment analysis of DEGs

**Figure 4:**
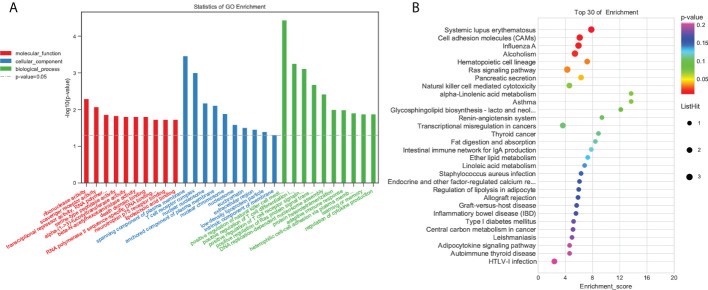
Functional enrichment analysis of DEGs. **(A)** GO enrichment. BP: Biological process. CC, Cellular component; MF, Molecular function. **(B)** KEGG pathway DEG enrichment scatter plot.

To understand the functions of the DEGs, we performed GO enrichment analysis. Enrichment analysis revealed that the DEGs were significantly enriched in immune-related GO terms (**Figure 4A**; **Table S3**). The significant terms included those involved in the adaptive immune response, immune system process, defense response, and innate immune response. KEGG enrichment analysis showed that DEGs were significantly enriched in the intestinal immune network for IgA production, PPAR signaling pathway, inflammatory bowel disease (IBD), ECM-receptor interaction, NF-kB signaling pathway, MAPK signaling pathway, and inflammatory mediator regulation of TRP channels (**Figure 4B**; **Table S4**).

### qRT–PCR validation

**Figure 5:**
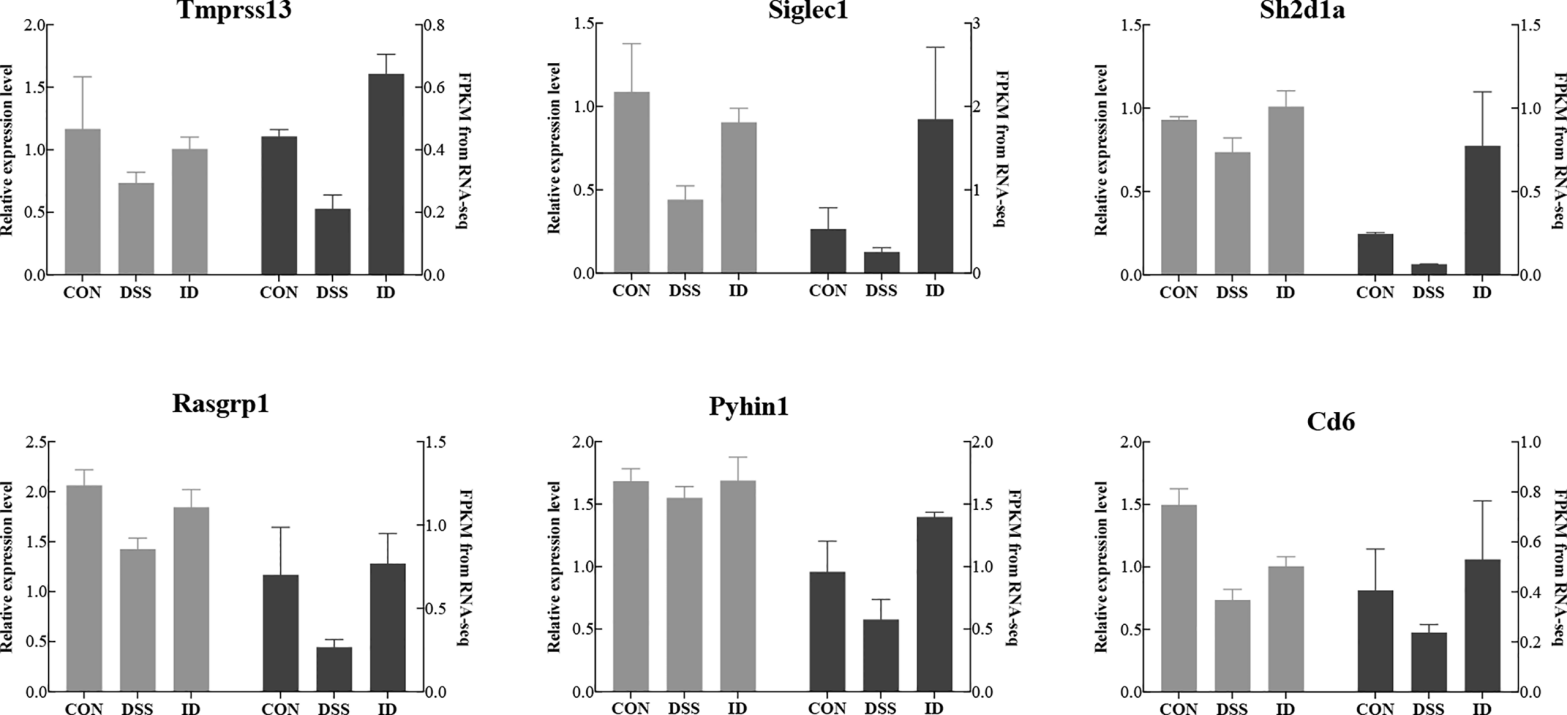
Comparison of the gene expression levels of RNA-seq with real-time PCR. The right axis represents the expression levels determined by RNA-seq in FPKM units, and the left axis represents gene expression levels determined by real-time PCR. Bars represent the mean ± SEM of three samples. The black column indicates the FPKM value; the gray column indicates the real-time PCR value using β-actin as a reference gene.

To verify the accuracy of the RNA-seq data and detect DEGs, we selected six DEGs (*Tmprss13, Siglec1, Rasgrp1, Pyhin1* and *Cd6*) for qRT-PCR analysis. Both RNA-seq and qPCR data showed same gene expression trends, suggesting RNA-seq accurately quantified colon gene expression (**Figure 5**).

### Effect of IPA on the gut microbiota under DSS-induced inflammation in the colon

**Figure 6:**
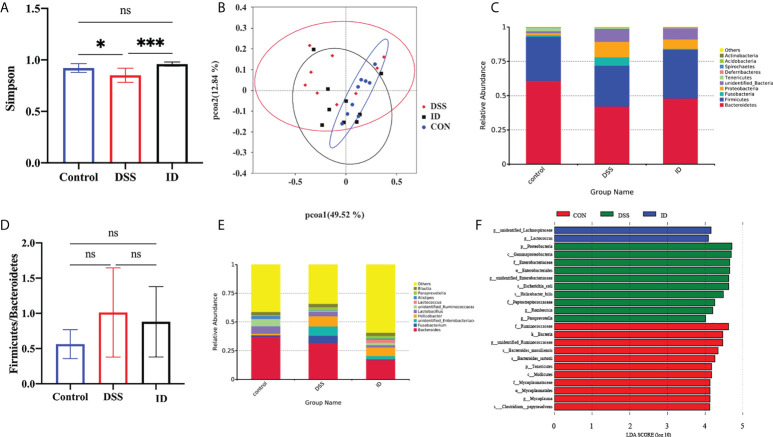
IPA modulated the composition of the gut microbiota in DSS-treated mice. **(A)** Index-group Difference Test of Simpson index in Sample Hierarchical Cluster Tree alpha-Diversity. Classification level: OTU. **(B)** Graph of principal component analysis (PCoA) at the genus level. **(C)** Microbial community bar plot at the phylum level with the relative abundance within the top 10. **(D)** Microbial community bar plot at the genus level with the relative abundance within the top 10. **(E)** Ratio of the percentage of 16S rRNA gene sequences assigned to Firmicutes versus Bacteroidetes. **(F)** Latent Dirichlet allocation (LDA) score distribution histogram and cladogram. P<0.05(*), P<0.05 (*), P<0.01 (***).

Next, we further explored the impact of IPA on the colonic microbiota composition of DSS-induced mice *via* 16S rRNA gene sequencing. The Simpson index was used to characterize the overall microbial diversity (**Figure 6A**). Beta diversity was analyzed using PCoA based on weighted_UniFrac, which revealed differences in the microbiota between the three groups (**Figure 6B**). The bacteria *Bacteroidetes* and *Firmicutes* were predominant at the phylum level, accounting for over 80% of the total microbial composition (**Figure 6C**). Moreover, IPA pretreatment displayed a trend toward a decreased *Firmicutes*/*Bacteroidetes* (F/B) ratio (**Figure 6E**). At the genus level of the microbiota in the colon, the relative abundance level of *Paraprevotella was* increased in the DSS group compared to the CON group, whereas it was decreased in the ID group compared with the DSS group (**Figure 6D**). The results showed that mice in the DSS group have reduced colonic microbial diversity, and IPA pretreatment alleviated these changes. To further identify specific bacterial taxa that significantly differentiate between the three groups, LEfSe analysis was applied and showed that *Lactococcus* was enriched in the ID group at the genus level (**Figure 6F**).

### Gene expression profile changes correlated with microbiota alterations in the colon

**Figure 7:**
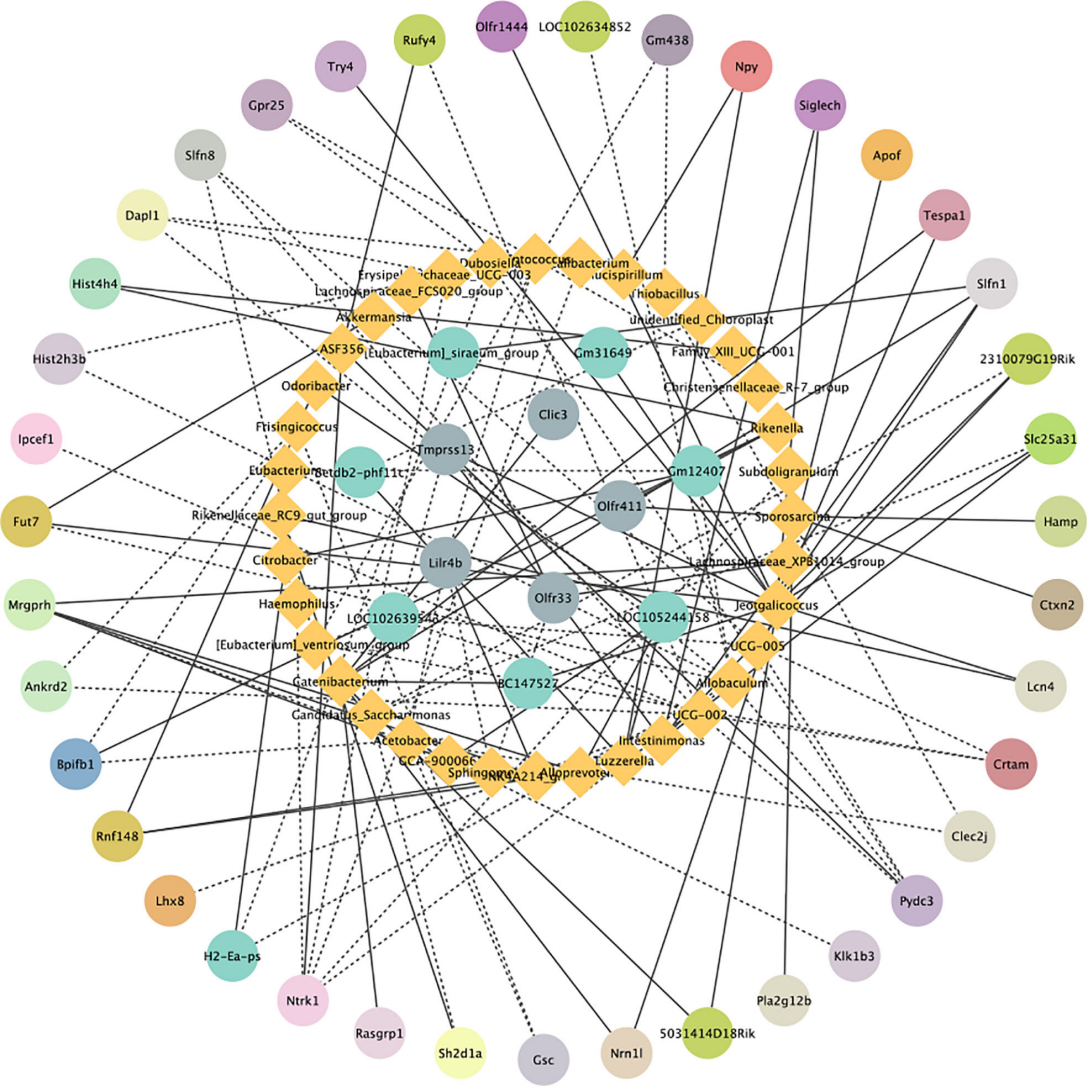
Correlation analysis graph of differentially expressed genes and microorganisms. Blue represents microorganisms and other colors represent enriched DEGs in the same pathway. The solid lines indicate a positive correlation and the dashed line indicates a negative correlation.

To investigate the relationship between gut microbiota and colonic gene expression, we performed Spearman correlation analysis. STEM analysis showed that 72 DEGs were significantly enriched in 6 profiles. Spearman’s correlation analysis of beneficial bacteria genus-level abundances and colonic genes is shown in **Figure 7**. Our findings demonstrated that the relative abundance levels of *Candidatus_Soleaferrea*, *Alloprevotella* and *Catenibacterium* were positively correlated with the expression levels of *Ermap*, *Mrgprh* and *Rasgrp1* in the colon, respectively (r=0.9412, *P*=0.0001; r=0.8984, *P*=0.0009; r=0.8645, *P*=0.0026). However, the relative abundances of *Candidatus_Saccharimonas* and *Acetobacter* were negatively correlated with the expression levels of *Sh2d1a* and *Bpifb1* in the colon, respectively (r =- 0.8645, *P* = 0.0026; r = -0.8660, *P* = 0.0025).

## Discussion

IBD, a common chronic inflammatory disease, has received great attention in recent years with an increasing global incidence (28, 29). Despite progress in its prevention, its impact continues to be being relevant, and new preventive strategies for IBD are needed (30, 31). Several tryptophan metabolites have been identified to protect against inflammation caused by IBDs (32). IPA is a deamine product of tryptophan that can only be produced by gut bacteria (33, 34). A growing body of evidence suggests that IPA plays an important regulatory role in the pathogenesis of a variety of diseases. However, it is not clear whether IPA has a potential effect on IBD prevention. The DSS-induced IBD model has been extensively used in the study of IBD pathogenesis (35). In this study, we first investigated the effect of IPA on the development of DSS-induced colitis in mice. To investigate the ameliorative effect of IPA on colitis inflammation in mice, we established a DSS- induced acute colitis murine model. Weight loss, shortened colonic length, and increased colonic weight are commonly used as indicators of the severity of IBD (36). IBDs are characterized by body weight loss, bloody feces, diarrhea, shortening of the colon length, and splenomegaly. Herein, our results showed that IPA treatment reversed the weight loss and shortening of the colon in the DSS-induced IBD mice and restored the destruction of colon epithelium and mucosa in the colon. Previous studies have also shown that tryptophan metabolites are effective in alleviating colitis in DSS-induced mice (37). Similar efficacy was shown in a model of high-fat diet (HFD)-fed rats (12). Collectively, these results demonstrate that IPA (200 mg/kg) effectively suppressed colonic inflammation in the DSS-induced IBD mouse model.

Cytokines are classified by their nature as proinflammatory and anti-inflammatory factors, and are commonly used as biomarkers of colonic inflammation and are correlated with disease severity (38). There is now evidence that the imbalance between pro- and anti-inflammatory cytokines in IBD patients hinders the resolution of inflammation (39). Therefore, modulation of cytokine levels is considered a potential strategy for the treatment of IBD. In regulating immune homeostasis, targeting inflammatory factors not only promotes the production of anti-inflammatory factors, but also reduces the amount of associated proinflammatory factors. Previous studies have found that IPA may be an important biomarker and renoprotective agent (40). Here, we further investigated the effect of IPA on inflammatory cytokine levels in DSS-induced colitis. The study indicated that, a large number of proinflammatory factors (IFN-γ, IL-1β, IL-6, and TNF-α) were produced in the serum and colonic tissues after colitis was induced by DSS in mice, while the anti-inflammatory factors (IL-10 and IL-4) decreased. After pretreatment with IPA, inflammation was alleviated, the proinflammatory factors IFN-γ, IL-1β, IL-6, and TNF-α were suppressed, and anti-inflammatory factor IL-10 and IL-4 were increased. Notably, IL-6 is a proinflammatory and anti-inflammatory factor that is a key mediator of many chronic and acute inflammatory responses (41). Low IL-6 concentrations enhance the body’s immune defense response (42).

Studies have proven that IgA is closely related to intestinal inflammation and can be secreted at mucosal sites in response to local inflammation. Previous studies have not examined the effects of IPA on IgA. In this study, the serum and colon contents of IgA were significantly decreased by IPA pretreatment compared with the untreated DSS group (*P <*0.05), which may be one of the reasons for the improved colitis symptoms. Similar protective effects against LPS-induced colitis were also observed; IPA protects LPS-induced mice by activating AhR to promote IL-10 production while suppressing the gene expression of TNF-α (43). Largely consistent with our results, similar reports of the expression profile of cytokines have been found in other models of DSS-induced colitis in mice (44). Sári et al. also identified reported that IPA exerts its antineoplastic modulation through the aryl hydrocarbon receptor (AHR) and pregnane X receptor (PXR) (45). Therefore, it is reasonable to assume that the ameliorative effect of IPA on DSS-induced colitis in mice may be related to the maintenance of inflammatory cytokine balance.

To elucidate the potential molecular mechanisms by which IPA treatment prevents DSS-induced colitis, we sequenced the colon’s transcriptome to analyze the biological functions and pathways of DEGs. STEM analysis revealed that IPA regulates the expression trends of DSS-induced colonic genes. Furthermore, inflammation-related terms were predominantly enriched in GO biological processes. Notably, KEGG analysis showed a significant association of inflammatory diseases such as the PPAR signaling pathway, inflammatory bowel disease (IBD), ECM-receptor interaction, NF-kappa B signaling pathway, MAPK signaling pathway, and inflammatory mediator regulation of TRP channel pathways. Among them, the MAPK signaling pathway regulates inflammation and immunity in the gut (46). A previous study revealed that acetic acid-induced colitis activates the MAPK pathway, and MAPK inhibitors were shown to decrease inflammation and specifically improve colitis in IBD animals (47). Studies have shown that DSS-induced acute colitis is mediated by the innate immune response and adaptive immunity contributes to the healing process of colitis (48). Our sequencing results also showed that DEGs were involved in the MAPK signaling pathway. Therefore, a systemic study of the MAPK signaling pathway can help further elucidate the development of IBD and provide new targets for preventing and treating of IBD.

The gut microflora is an important regulator of intestinal homeostasis (49, 50). Numerous studies have shown that microbial imbalance may also result in a variety of diseases and immune responses (51, 52). The ratio of Firmicutes/Bacteroidetes (F/B) is an important index of gut microbiota structure change (53). Our study revealed that *Bacteroidetes* and *Firmicutes* were the most abundant phyla in the colonic contents of mice. Several studies have observed an increase in the F/B ratio in a DSS-induced colitis mouse model (54). In addition, the increase in the abundance of *Proteobacteria* is considered a dysbiosis of the gut microbiome (55). Consistent with previous results, the DSS group had a higher ratio of *Firmicutes* to *Bacteroidetes* and a higher relative abundance of *Proteobacteria* as compared to the CON group. However, IPA pretreatment reversed these DSS- induced changes. The *Lactobacillus* genus is a well-known probiotic group with colitis-alleviating effects in *in vivo* mouse models (56, 57). Researchers have also found that *Lactococcus* plays a role in maintaining gut flora balance and the prevention of pathogenic invasion. Similarly, LEfSe demonstrated that at the genus level *Lactococcus* was enriched in the ID group, which might explain the effect of IPA in alleviating colitis. These results indicate that IPA administration alleviates DSS-induced colitis by restoring the gut microbiota composition.

Finally, we used Spearman correlation analysis to determine the relationship between gut microbiota and host colon gene expression. It is worth noting that we found that *Candidatus_Soleaferrea, Alloprevotella* and *Catenibacterium* were positively correlated with gut immune gene expression, while *Candidatus_Saccharimonas* and *Acetobacter* were negatively correlated with gut barrier gene expression. This is clearly indicative of potential relationships between gut microbiota and gut functions, which should be investigated further in future targeted studies. Recent observations suggest that the gut microbiota may affect the progression of colitis by modulating the host immune response (58). *Alloprevotella* was positively related to *Mrgprh*. *Prevotella, Phascolarctobacterium*, and *Catenibacterium*, as producers of short-chain fatty acids (SCFAs), play a key role in intestinal homeostasis and are thought to be beneficial to host health (59, 60). *Slfn1* is a late LPS response gene in mouse macrophages (61). Moreover, we also found that the relative abundance of *Catenibacterium* showed was significantly and positively correlated with the mRNA level of *Slfn1* in the colon, suggesting a potential regulatory interaction between *Catenibacterium* and *Slfn1*. These results suggest that IPA suppresses the immune response by regulating colon gene expression, which relies on gut microbiota.

## 5 Conclusions

We determined whether IPA can alleviate DSS-induced colitis symptoms and its potential mechanism. In this study, IPA pretreatment ameliorated DSS-induced intestinal damage and decreased proinflammatory cytokine contents in serum and colon tissue. Meanwhile, IPA regulated the diversity and composition of the colonic microbiota, and modulated gene expression, which enhanced the immune response. These findings should provide a theoretical foundation for using IPA as a nutritional intervention to improve animal intestinal health and nutrition.

## Data availability statement

The datasets presented in this study can be found in online repositories. The names of the repository/repositories and accession number(s) can be found below:

https://www.ncbi.nlm.nih.gov/, PRJNA857541

https://www.ncbi.nlm.nih.gov/, PRJNA860780.

## Ethics statement

The animal study was reviewed and approved by the Animal Welfare Committee of the Institute of Subtropical Agriculture, Chinese Academy of Sciences, Changsha, China.

## Author contributions

KX designed the experiments. YF, HG, XH, and YC performed the experiments. HG, XH and YC processed the data. YF wrote the original draft. KX revised the paper. All authors have read and agreed to the published version of the manuscript.

## Funding

This work was supported by the Laboratory of Lingnan Modern Agriculture Project (NT2021005), the Special Funds for the Construction of Innovative Provinces in Hunan (2021NK1009, 2021NK1012, 2020WK2030, and 2020JJ5635), the Natural Science Foundation of Guangxi Province (2020JJB130030) and the Open Fund of Key Laboratory of Agro-ecological Processes in Subtropical Region, Chinese Academy of Sciences (ISA2019304).

## Conflict of interest

The authors declare that the research was conducted in the absence of any commercial or financial relationships that could be construed as a potential conflict of interest.

## Publisher’s note

All claims expressed in this article are solely those of the authors and do not necessarily represent those of their affiliated organizations, or those of the publisher, the editors and the reviewers. Any product that may be evaluated in this article, or claim that may be made by its manufacturer, is not guaranteed or endorsed by the publisher.
